# MIKC^C^-type MADS-box genes in *Rosa chinensis*: the remarkable expansion of ABCDE model genes and their roles in floral organogenesis

**DOI:** 10.1038/s41438-018-0031-4

**Published:** 2018-05-01

**Authors:** Jinyi Liu, Xiaodong Fu, Yuwei Dong, Jun Lu, Min Ren, Ningning Zhou, Changquan Wang

**Affiliations:** 1 College of Horticulture, Nanjing Agricultural University, Nanjing, Jiangsu 210095 China; 2 Flower Research Institute, Yunnan Academy of Agricultural Sciences, Kunming, Yunnan 650200 China

## Abstract

MIKC^C^-type MADS-box (MIKC^C^) genes encode transcription factors that have crucial roles in controlling floral organogenesis and flowering time in plants. Although this gene family has been well characterized in many plant species, its evolutionary and comprehensive functional analysis in rose is lacking. In this study, 58 non-redundant MIKC^C^ uni-transcripts were extensively identified from rose transcriptomes. Phylogenetic analysis placed these genes into 12 clades with their *Arabidopsis* and strawberry counterparts, and revealed that ABCDE model (including AP1/FUL, AP3/PI, AG, and SEP clades), and SOC1 and AGL6 clade genes have remarkably expanded in *Rosa chinensis*, whereas genes from the FLC and AGL17 clades were undetectable. Sequence alignments suggest that the AP3/PI clade may contribute to more specific functions in rose due to a high variation of amino acid residues within its MADS-box domains. A comparative analysis of gene expression in specific floral organ differentiation stages and floral organs between *R. chinensis* cv. Old Blush and the closely related mutant genotype *R. chinensis* cv. Viridiflora (floral organs mutated into leaf-like structures) further revealed the roles of ABCDE model genes during floral organogenesis in rose. Analysis of co-expression networks provided an overview of the regulatory mechanisms of rose MIKC^C^ genes and shed light on both the prominent roles of AP3/PI clade genes in floral organogenesis and the roles of *RcAGL19*, *RcAGL24*, and *RcSOC1* in regulating floral transition in rose. Our analyses provide an overall insight of MIKC^C^ genes in rose and their potential roles in floral organogenesis.

## Genetics: How to build a rose

Roses have been prized throughout history for their beauty and now we’re closer to understanding the genetics underpinning their appearance. MIKC^c^ genes encode transcription factors and have long been implicated in flower organ development; they fall into five classes, the precise combination of which underpins the type of flower that develops. Changquan Wang at Nanjing Agricultural University in China and colleagues compared MIKC^c^ gene transcripts from roses with those from *Arabidopsis* and strawberries, identifying certain genes that were remarkably expanded, and others that were missing. They also compared MIKC^c^ genes from Old Blush roses with those from their mutant relative Viridiflora, pinpointing specific genes which appeared altered. Further characterization of such genes may enable a better understanding of why rose flowers differ and aid the design of even more beautiful varieties.

## Introduction

MADS-box genes, which are defined by the presence of a highly conserved DNA-binding MADS-box domain in the N-terminal region of a protein sequence, encode a family of transcription factors (TFs) that are involved in various aspects of plant growth and developmental processes, especially floral organogenesis and flowering time^1–3^. Based on phylogenetic analysis of MADS-box genes in eukaryotes, two major lineages, termed type I and type II, were distinguished^1^. MIKC^C^-type MADS-box (MIKC^C^) genes are plant specific and some, which were initially and are best known as floral organ identity genes, comprise the majority of genes from the type II lineage^4,5^. In the model plant *Arabidopsis thaliana*, MIKC^C^ genes can be phylogenetically clustered into 12 clades, i.e., AP1/FUL, AP3/PI, AG, SEP, SOC1, SVP, AGL6, AGL12, AGL15, AGL17, FLC, and BS clades, along with their counterparts from other plants^6,7^. According to the well-known ABCDE model or ‘floral quartets’ model of floral development^8,9^, the floral organ identity genes, which are mainly derived from the AP1/FUL, AP3/PI, AG, and SEP clades of the MIKC^C^ gene family, can be further divided into five different classes, termed class A (corresponding to the AP1 genes from the AP1/FUL clade), B (AP3/PI), C (AG), D (STK genes from the AG clade), and E (SEP) genes, based on their different homeotic functions in floral organogenesis. In this model, A refers to sepals, A + B + E refers to petals, B + C + E refers to stamens, C + E refers to carpels, and D (sister clade of the C class) refers to ovules^5,10^. ABCDE model genes have been functionally characterized, mainly through homeotic flower mutants^11^, including the celebrated homeotic mutants from *A. thaliana* and *Antirrhinum majus*^5,12^. Loss of function or ectopic expression of A, B, C, D, and E genes usually leads to homeotic conversion of floral organs and floral aberrations^2,3,12–15^. For example, the loss of SEP clade genes in *sep* quadruple mutants (*sep1/2/3/4*) can turn the flower into leaf-like structures^16,17^, whereas ectopic expression of *AG* genes can lead to partial homeotic transformations of the sepals and petals toward carpels and stamens, respectively^13^. In addition, among the floral organ identity genes, several crucial MIKC^C^ genes have been found to modulate flowering time in *Arabidopsis*^2,3,18–23^. Specifically, *FLC*, *SOC1*, *AGL19*, and *AGL24* genes have been found to be vital components of the vernalization pathway^23^. Nevertheless, the regulatory mechanisms of MIKC^C^ genes in regulating flowering transition and floral organogenesis have not been fully elucidated.

MADS-box genes are believed to have originated from gene duplications that initially occurred in the lineage of the most recent common ancestor of extant eukaryotes^24^. Thus far, MADS-box genes have been identified in nearly all groups of eukaryotes, including plants, animals, and fungi^1^. However, the number and functional diversity of MADS-box genes appear to have increased remarkably during the evolution of land plants, especially flowering plants (angiosperms), with more than 100 genes in *Arabidopsis* (*A. thaliana*), rice (*Oryza sativa*), and poplar (*Populus trichocarpa*)^7,25–27^. The expansion and functional diversification of plant MADS-box genes are tightly linked to the evolution of plant body plans and life history strategies^1,3,28–30^. Novel MADS-box genes and/or new functions have typically arisen in specific lineages of plants or even specific species^3^ and are closely linked to the evolution of novel structures (such as seeds, flowers, and fruits) and habitat-specific adaptations^31,32^. For instance, most subclades of MIKC^C^ genes appear to have originated from ancestral seed plants^1^. In addition, within this group, the AP1/SQUA and SEP subclades seem to be the first to have duplicated or expanded in flowering plants, suggesting their key roles in the origination and subsequent morphological evolution of flowers^1,3,33^. In addition, six *DORMANCY-ASSOCIATED MADS-BOX* (*DAM*) genes that originated from a series of tandem duplications in peach (Rosaceae) have been associated with floral bud dormancy and thereby seasonal flowering^34^. Contrarily, multiple losses of MIKC^C^ genes have also been identified, especially in TM8-like and FLC-like gene clades that might have been adaptive during plant evolution^35^. As a consequence of the frequent expansion, and gene loss and diversification of the MADS-box gene family, a full understanding of the biological functions of MADS-box genes in flower development will not only require understanding of the regulatory mechanisms of the MADS-box genes but will also require their identification and functional characterizations in a variety of species.

Roses (genus *Rosa*), which belong to the large Rosaceae family^36^, are one of the most commonly cultivated ornamental plants worldwide and are highly popular as cut flowers with a wide range of flower types, fragrances, and colors. The continuous flowering (CF) trait of *Rosa chinensis* implies differences in its flowering mechanisms and may subsequently lead to differences in floral organ development, as these are closely related proceses^36,37^. Although a few orthologs of the MIKC^C^ genes, such as *RhAPETALA3* (*RhAP3*), *RhPISTILLATA* (*RhPI*), *RhAGAMOUS* (*RhAG*), and *RhSHATTERPROOF*, which are mainly involved in floral organogenesis, have been isolated from *Rosa hybrida* cultivars and functionally characterized to some extent^38–43^, a comprehensive understanding of the role of MIKC^C^ genes in controlling rose flowering transition and floral organogenesis at the genome level is still lacking.

Commonly known as the “green rose,” *R. chinensis* cv. Viridiflora is a homeotic flower mutant of Chinese old rose^44^, with the floral organs mutated into leaf-like structures (called ‘phyllody’) instead of true petals, stamens, and pistils^45^ (Supplementary File S1). Similar phenotypes have also been observed in *Arabidopsis*^16,17^ and *Catharanthus roseus*^46,47^—these phenotypes can be caused by quadruple mutants (*sep1/2/3/4*) of SEP clade genes or by phytoplasma infection. However, both of these potential causes of phyllody were contradicted by the research of Yan et al.^48^, in which reciprocal grafting experiments between Viridiflora and *R. chinensis* cv. Old Blush did not cause the phyllody phenotype and expressions of *RcSEP1* and *RcSEP3* were detected. Thus, the mechanism of floral organogenesis of Viridiflora remains a mystery. In addition to having an interesting floral phenotype, *R. chinensis* cv. Viridiflora is closely related to *R. chinensis* cv. Old Blush^48^ (Supplementary File S1), which is a very important common ancestor of commercial modern roses and is the original source of the CF and tea scent traits that exist in modern roses. *R. chinensis* cv. Old Blush is also widely used in many breeding programs and has been suggested as a model cultivar for rose research^36,43^. These two closely related genotypes provide an excellent system for studying the homeotic functions of MIKC^C^ genes in the floral organogenesis of rose.

In our study, a comprehensive identification of MIKC^C^ genes was performed based on high-quality transcriptome datasets for rose (two datasets were previously produced by our group)^43,48–50^. Phylogenetic analysis revealed the remarkable expansion of rose ABCDE model genes, whereas FLC and AGL17 clade genes were not detected. Comparative gene expression analyses between Old Blush and its homeotic flower mutant Viridiflora across the major floral organ differentiation stages and different floral organs confirmed the roles of MIKC^C^ genes in rose floral organogenesis. Co-expression networks of MIKC^C^ genes were generated from transcriptome data, revealing the prominent roles of AP3/PI clade genes in floral organogenesis and of *RcAGL19*, *RcAGL24*, and *RcSOC1* in regulating floral transition in rose. Our analyses provide a fundamental framework for the biological functions of MIKC^C^ genes in rose floral organogenesis and flowering transition, and will facilitate the functional characterization of individual MIKC^C^ genes in rose.

## Results

### Transcriptomic identification of MIKC^C^ genes in rose

After extensively searching the integrated transcriptome database with two different HMM profiles of the MADS-box domain (see Materials and Methods section), a total of 58 non-redundant uni-transcripts were assigned as rose MIKC^C^ genes (Supplementary File S2). This number is 1.49 (58/39) times and 1.38 (58/42) times higher than that in *Arabidopsis* and strawberry, respectively, indicating that MIKC^C^ genes have expanded in rose. The rose MIKC^C^ genes were assigned names consistent with *Arabidopsis* MIKC^C^ gene names, based mainly on their phylogenetic relationships (Supplementary File S2). As the draft genome sequence of a wild rose, *Rosa multiflora* (once-flowering), was very recently released^51^, we searched the best hit orthologs for the MIKC^C^ genes we identified from *R. chinensis* (CF). Based on the E-value and careful checking of the sequence similarity, we believe that some obvious differences exist in the MIKC^C^ gene family between those two species. For example, *RcAP1.1*, *RcAP1.2*, *RcAGL24.2*, *RcAGL6.1*, *RcAGL6.2*, and *RcAGL104.2* have low E-values with respect to their best hit in *R. multiflora*, suggesting they might be genes specific to *R. chinensis* (Supplementary File S2). In addition, reciprocal best hit orthologs of rose MIKC^C^ genes were identified in *Arabidopsis* and rice (*O. sativa*). Eighteen and 14 rose MIKC^C^ genes were detected to have their reciprocal best hit orthologs in *Arabidopsis* and rice, respectively, suggesting that these genes might be functionally conserved between plant species. Furthermore, the highest count number and fragments per kilobase of transcript per million mapped reads (FPKM) values from the currently available RNA sequencing (RNA-seq) data were presented to provide an overview of the expression levels of the rose MIKC^C^ genes identified in our study (Supplementary File S2).

### Phylogenetic analysis of rose MIKC^C^ proteins

**Fig. 1:**
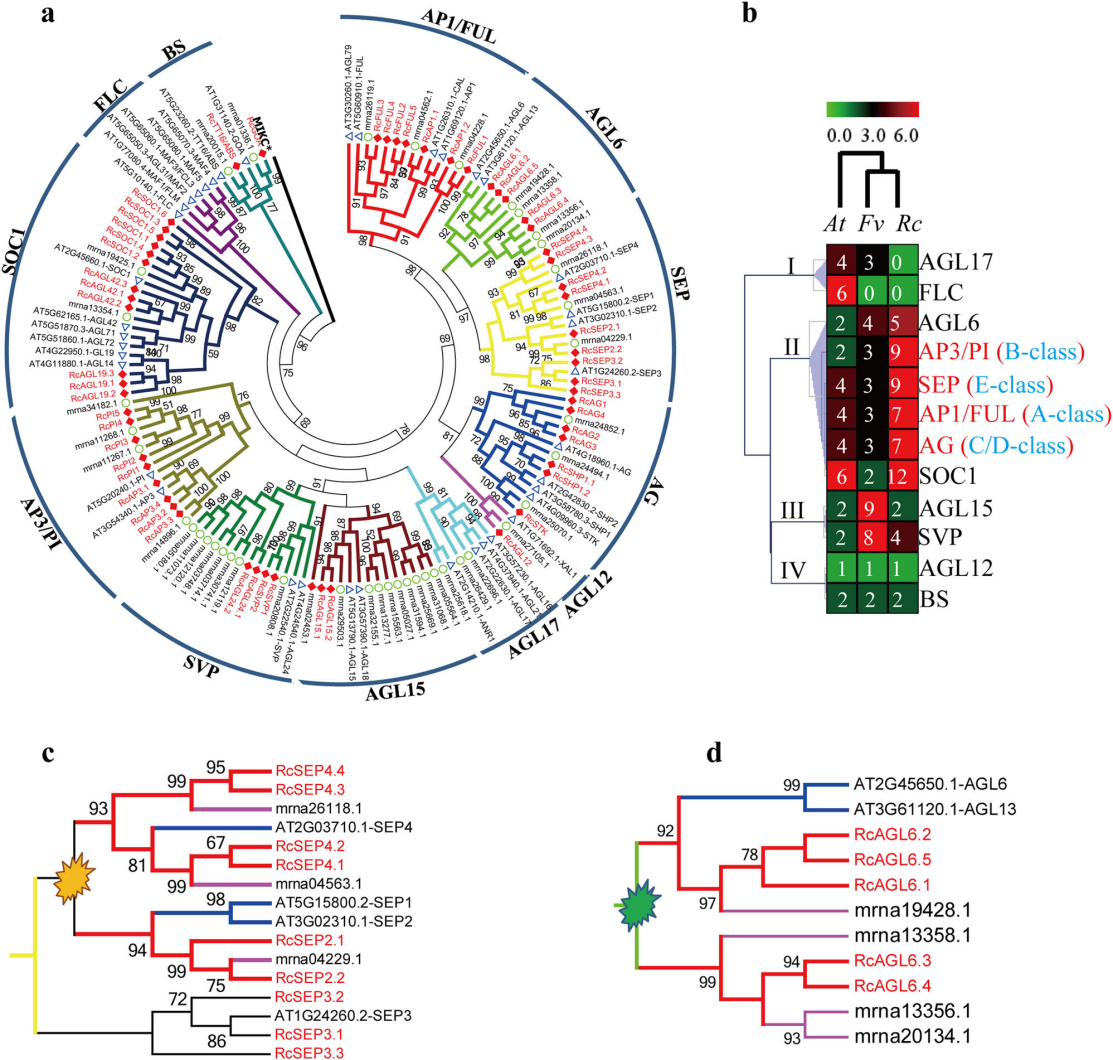
Phylogenetic analysis of MIKC^C^ genes from rose, *Arabidopsis* and strawberry. **a** Maximum likelihood (ML) phylogenetic trees of rose, *Arabidopsis* and strawberry MIKC^C^ proteins (see Materials and Methods section for the parameters of tree construction). **b** Heatmap of the number of different clade genes from rose, *Arabidopsis* and strawberry. I*–*IV denote different clusters and the numbers in the colored box denote the number of MIKC^C^ genes. *At*, *A. thaliana*, *Fv*, *Fragaria vesca*, *Rc*, *R. chinensis*. **c**,** d** Gene duplication events of the MIKC^C^ gene family detected based on the phylogenetic relationships among rose, *Arabidopsis* and strawberry. The orange explosive shape denotes the gene duplication event that might have occurred before the split of *Arabidopsis* and rosaceae, whereas the green one denotes the gene duplication event that might have occurred within rosaceae

To examine the phylogenetic relationships among MIKC^C^ proteins and to group them within established clades, a phylogenetic tree was constructed for MIKC^C^ proteins from *Arabidopsis*, strawberry, and rose using MAFFT v7.037b with the specific sequence alignment parameter (E-INS-I) (see Materials and Methods section) (Fig. 1a). All the proteins were phylogenetically classified into 12 clades along with the established clades in *Arabidopsis* (the MIKC* were placed as the root branch), which included the AP1/FUL, AP3/PI, AG, SEP, SOC1, SVP, AGL6, AGL12, AGL15, AGL17, FLC, and BS clades. Interestingly, there is no rose MIKC^C^ protein classified into the AGL17 clade present in *Arabidopsis* and strawberry, and neither rose nor strawberry has genes in the FLC clade (Fig. 1b, I cluster), indicating that these clades might have suffered extensive gene loss during their evolution. In contrast, the numbers of MIKC^C^ proteins in eight clades, i.e., the AP1/FUL (7/4), AP3/PI (9/2), AG (7/4), SEP (9/4), AGL6 (5/2), and SOC1 (12/6) clades in rose (Fig. 1b, II cluster), and AGL15 (9/2) and SVP (8/2) in strawberry (Fig. 1b, III cluster), were much higher than their counterparts in *Arabidopsis*, indicating that these clades expanded remarkably after the split of *Arabidopsis* and rosaceae. By contrast, the number of AGL12 (1) and BS (2) clades (Fig. 1b, IV cluster) was the same in all three species we examined, suggesting that these clades could be functionally conserved in plants.

Examination of the phylogenetic relationships among MIKC^C^ proteins from *Arabidopsis*, strawberry and rose revealed 16 closely related paralogous gene pairs in rose, suggesting recent duplication events. Two obvious imprints of gene duplication events were detected in the phylogeny of MIKC^C^ proteins. One such event was detected in the SEP clade (Fig. 1c), from which two clades emerged containing MIKC^C^ proteins from *Arabidopsis*, strawberry, and rose, suggesting a gene duplication event that contributed to the occurrence of the two groups before the split of *Arabidopsis* and rosaceae. A second instance of gene duplication was found in the AGL6 clade (Fig. 1d), from which two clades emerged, but they contained only the MIKC^C^ proteins from strawberry and rose, suggesting that a gene duplication event may occurred within rosaceae and contributed to the two groups.

### The structural diversity of MADS-box domains of AP1/FUL, AP3/PI, AG, and SEP clade genes in rose

**Fig. 2:**
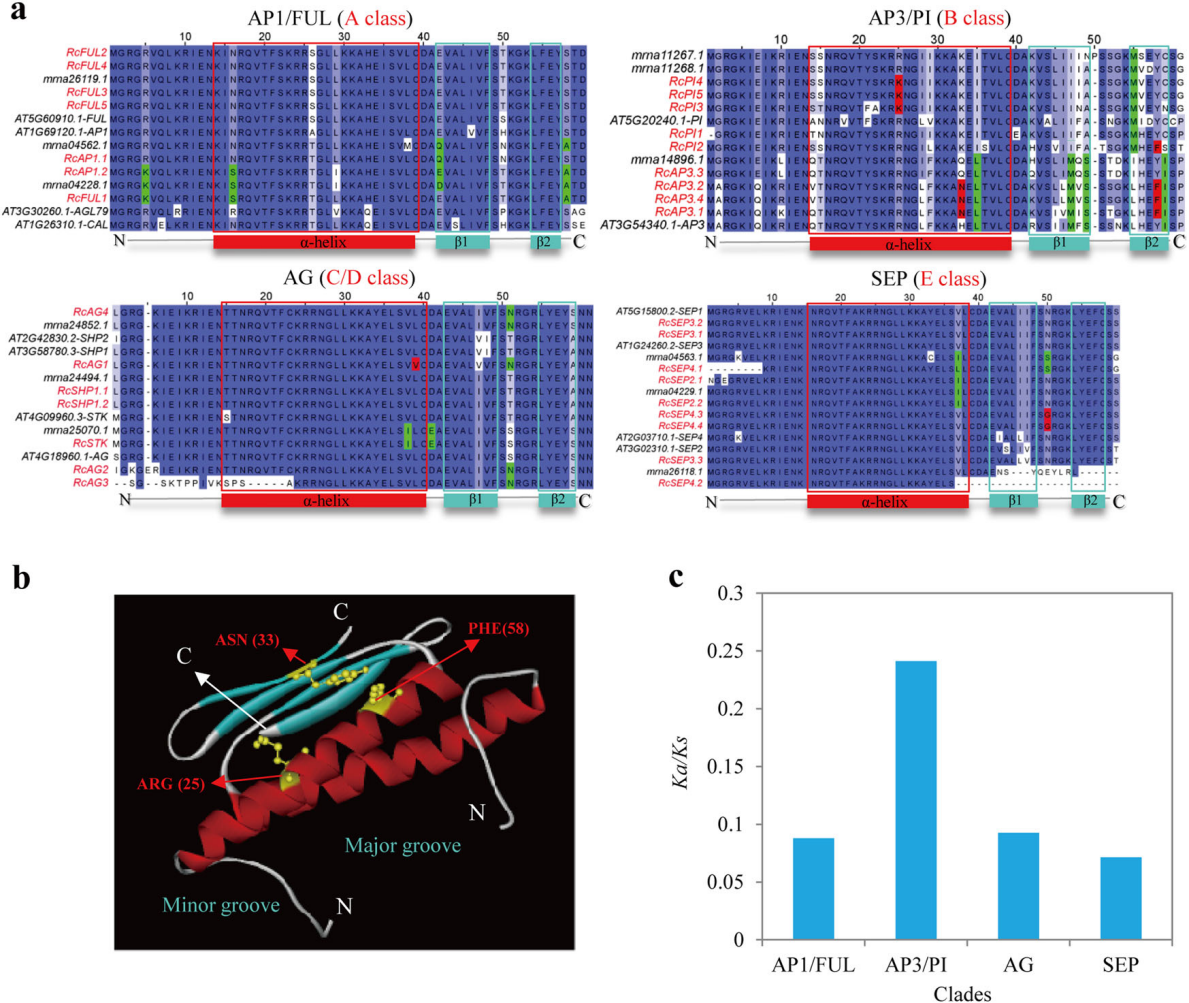
Structural features of MADS-box domain proteins of AP1/FUL, AP3/PI, AG, and SEP clades. **a** Sequence alignments of MADS-box domain proteins of the AP1/FUL, AP3/PI, AG, and SEP clades. The amino acid residues in the red boxes are rose specific, and those in the green boxes appear to be specific to rosaceae. **b** The homo-dimer tertiary structure of MADS-box domain proteins; RcPI4 is shown as an example. **c** *Ka*/*Ks* rates within the MADS domains of the AP1/FUL, AP3/PI, AG, and SEP clades

To gain more insights into the functional diversity of AP1/FUL, AP3/PI, AG, and SEP clade genes in rose, which correspond to the homology of A-, B-, C/D-, and E-class genes, respectively, in the ABCDE model, multiple sequence alignments of MADS-box domain proteins were generated for these clades (Fig. 2a) and a homo-dimer tertiary structure of MADS-box domain proteins (RcPI4 as an example) was constructed to provide an overview of their structural features (Fig. 2b). We found that the divergence rates of amino acid residues and the *Ka/Ks* value within MADS-box domains from AP3/PI clade genes (corresponding to the homology of B- class genes) were much higher than those from AP1/FUL, AG, and SEP clades across different species (Fig. 2c), suggesting that important functional divergence of AP3/PI clade genes may contribute to rose floral organogenesis and development. Furthermore, three amino acid residues in the MADS-box domains of AP3/PI clade genes, i.e., lysine (K) at site 25, asparagine (N) at site 33, and phenylalanine (F) at site 58, were found to be rose specific (Fig. 2a, b), suggesting that these sites could contribute to DNA-binding and dimerization properties of MADS-box TFs in rose. Furthermore, the homo-/heterodimeric states of all the MADS-box domain proteins from *Arabidopsis*, strawberry, and rose were predicted to provide more insight into their dimerization properties (Supplementary File S3).

### Expression patterns of rose MIKC^C^ genes in developmental processes

**Fig. 3:**
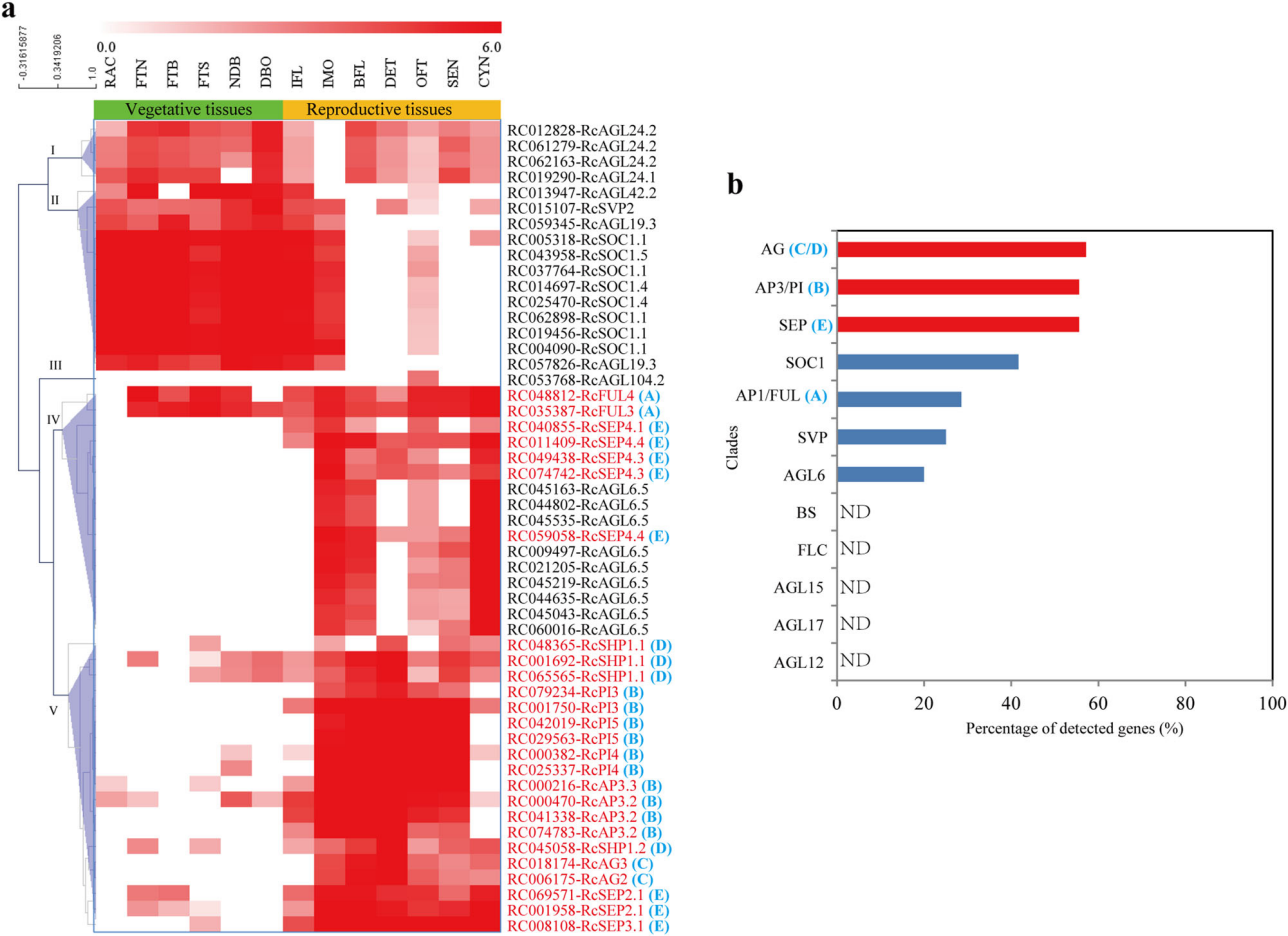
Expression patterns of rose MIKC^C^ genes in different tissues. **a** Heatmap of the expression patterns of rose MIKC^C^ genes. The heatmap was generated using MeV software v. 4.9.0 and is accompanied by a color scale corresponding to the gene expression level (FPKM values) from undetected (0.0) to ≥ 6. The sample names correspond to the previous descriptions^43^—BFL, closed flower; CYN, rose hip from pollination up to early pigmentation; DBO, active axillary buds (vegetative meristem); DET, stamens at microsporogenesis and microgametogenesis stages; FTB, leaves infected with *Botrytis cinerea* LR18; FTN, young leaves and stems; FTS, leaves from water-stressed plants; IFL, floral bud at floral meristem transition; IMO, floral meristem and early floral organ (sepals, petals, stamens and carpels) development; NDB, dormant axillary buds (vegetative meristem); OFT, open flower; RAC represents white young roots; and SEN, senescent flower. The black box marks the two most important stages (IFL and IMO) of floral transition. **b** The percentage of all MIKC^C^ genes detected in the transcriptome dataset of Dubois et al.^43^ The percentages were cumulated according to the total number (58) of the MIKC^C^ genes we identified in our study. The top three clades with a higher percentage of detected genes are highlighted in red

MIKC^C^ genes have been suggested to be involved mainly in the regulation of reproductive organ identity and flowering time in plants. To further associate their biological function with specific developmental processes in rose, we re-analyzed their expression patterns across all the typical organs (six vegetative and seven reproductive organs) of Old Blush using the public RNA-seq datasets contributed by Dubois et al.^43^. As shown in Fig. 3a, five major clusters of gene expression patterns were distinguished: cluster I, containing *RcAGL24.1* and *RcAGL24.2*, was expressed in all tissues except the floral meristem and early floral organs (IMO); cluster II, containing *RcAGL42.2*, *RcSVP2*, *RcAGL19.3*, *RcSOC1.1*, *RcSOC1.4*, and *RcSOC1.5*, was highly expressed in vegetative tissues, the floral meristem (IFL) and IMO but was reduced or nearly undetectable (ND) in the completely differentiated flower (BFL, DET, OFT, SEN) and rose hip from pollination to early pigmentation (CYN); cluster III, containing only one gene (*RcAGL104.2*), was detected only in open flowers (OFT); cluster IV, containing *RcFUL3*, *RcFUL4*, *RcSEP4.1*, *RcSEP4.3*, *RcSEP4.4*, and *RcAGL6.5*, was mainly characterized by high expression in the floral meristem, and IMO and CYN; and cluster V, containing *RcAP3.2*, *RcAP3.3*, *RcPI3, RcPI4, RcPI5, RcAG2, RcAG3*, *RcSHP1.1*, *RcSHP1.2*, *RcSEP2.1*, and *RcSEP3.1*, was characterized by high expression in the floral meristem, IMO, and the completely differentiated flower but was relatively reduced or ND in CYN. These gene expression patterns suggest the functional importance and diversification of rose MIKC^C^ genes in flowering transition and floral organogenesis.

To better understand the biological functions of MIKC^C^ genes in different clades, the number of detected MIKC^C^ genes in the transcriptome dataset of Dubois et al.^43^ was counted for all 12 clades (58 genes) that we identified from all the transcriptome datasets. Only 7 (58.3%) out of 12 clades were detected to contain MIKC^C^ genes from this dataset (Fig. 3b), and among them the proportion of detected MIKC^C^ genes in the AG, AP3/PI, and SEP clades ranked in the top three. These results may indirectly reflect the remarkable expansion of ABCDE model genes (Fig. 1b) and may provide an indication of the role of MIKC^C^ genes in floral organogenesis and development.

### The expression profiles of rose AP1/FUL, AP3/PI, AG, and SEP clade genes in floral organogenesis

**Fig. 4:**
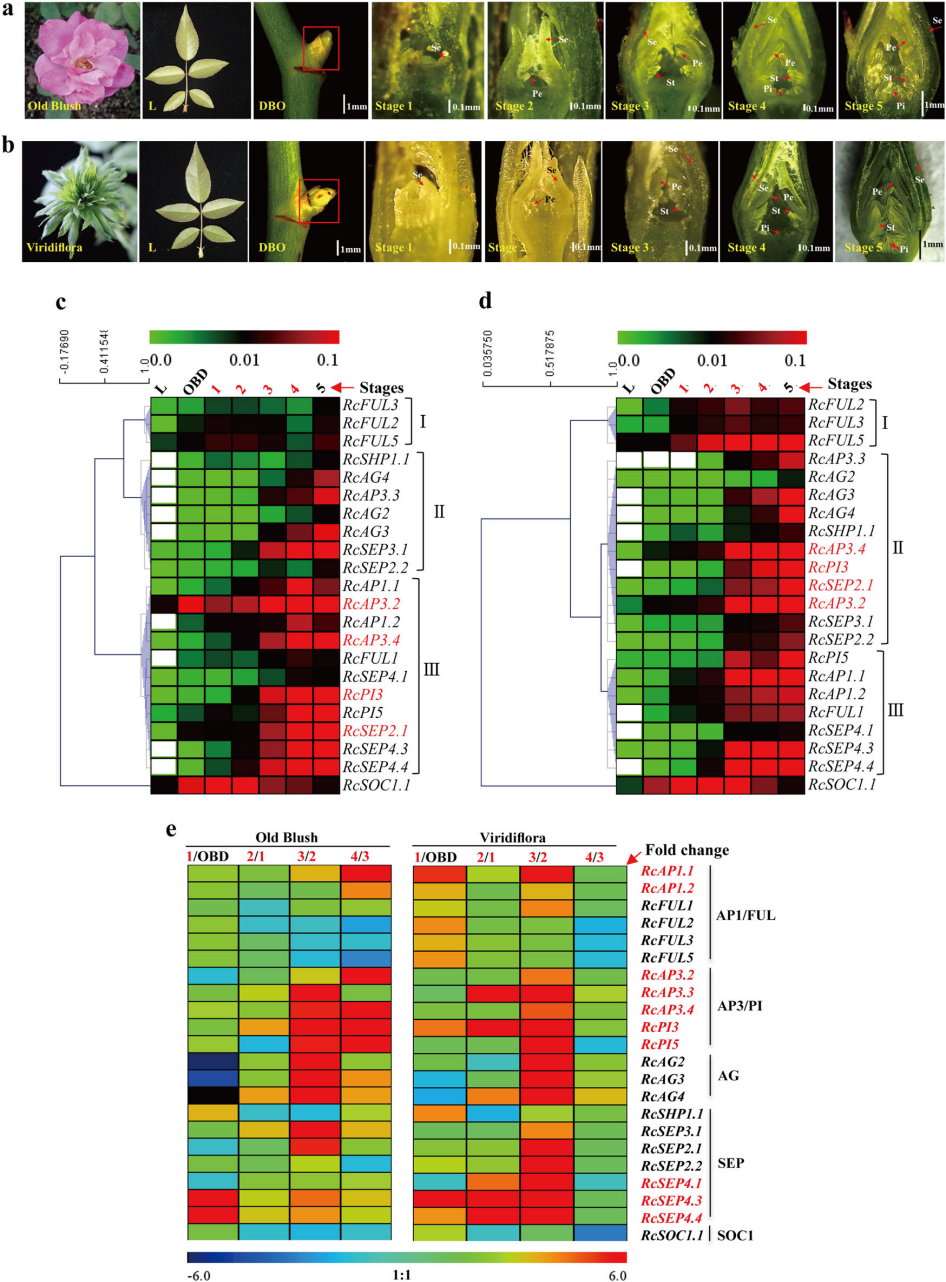
The expression profiles of rose MIKC^C^ genes from AP1/FUL, AP3/PI, AG, and SEP clades (ABCDE model genes) in response to floral organogenesis in *R. chinensis* cv. Old Blush and *R. chinensis* cv. Viridiflora. **a**,** b** Rose flower development stages of cultivars Old Blush and Viridiflora, respectively. L, leaves; DBO, vegetative meristem stage; Stage 1–4, initiation stages of sepals (stage 1), petals/petal-like (stage 2), stamens/stamen-like (stage 3), and carpels/carpel-like (stage 4) in Old Blush and Viridiflora; Stage 5, hypanthium starts to sink below the perianth and stamens; Se, sepals; Pe, petals; St, stamens; Pi, pistils. **c**,** d** Heatmaps of the relative gene expression levels of MIKC^C^ genes in *R. chinensis* cv. Old Blush and *R. chinensis* cv. Viridiflora, respectively, as determined by qRT-PCR. The relative gene expression levels were normalized with the *RcTUBULIN*, *RcGAPDH*, and *RcTCTP* reference genes that were used in previous studies^43,48^. **e** Fold change of gene expression for the sepal, petal, stamen, and pistil differentiation phases, which were calculated via comparison with the previous development phase

To compare the developmental difference between Old Blush and Viridiflora (in which petals, stamens, and pistils are converted into leaf-like organs and the flowers are fully sterile), the dissected structures of floral buds from different floral developmental stages were observed by stereo microscope (see Materials and Methods section). Six developmental stages for rose floral organogenesis were then defined in both the Old Blush (Fig. 4a) and Viridiflora (Fig. 4b) cultivar, including the active axillary bud stage (DBO, named according to the study by Dubois et al.^43^) and five morphologically distinct flower developmental stages (stage 1–5) named according to another study conducted by Dubois et al.^52^ Among these stages, DBO represents the vegetative meristem stage, whereas stages 1–4 represent the initiation of sepals (stage 1), petals/petal-like (stage 2), stamens/stamen-like (stage 3), and carpels/carpel-like (stage 4), respectively; in stage 5, all floral organs are apparent, and the hypanthium starts to sink below the perianth and stamens^52^. A comparison of the corresponding developmental stages between Old Blush and Viridiflora showed that while the order and timing of floral organogenesis between Old Blush and Viridiflora are very similar, the morphological structures of their floral organs are different, especially the morphological structures during the stamen initiation stage (stage 4).

**Fig. 5:**
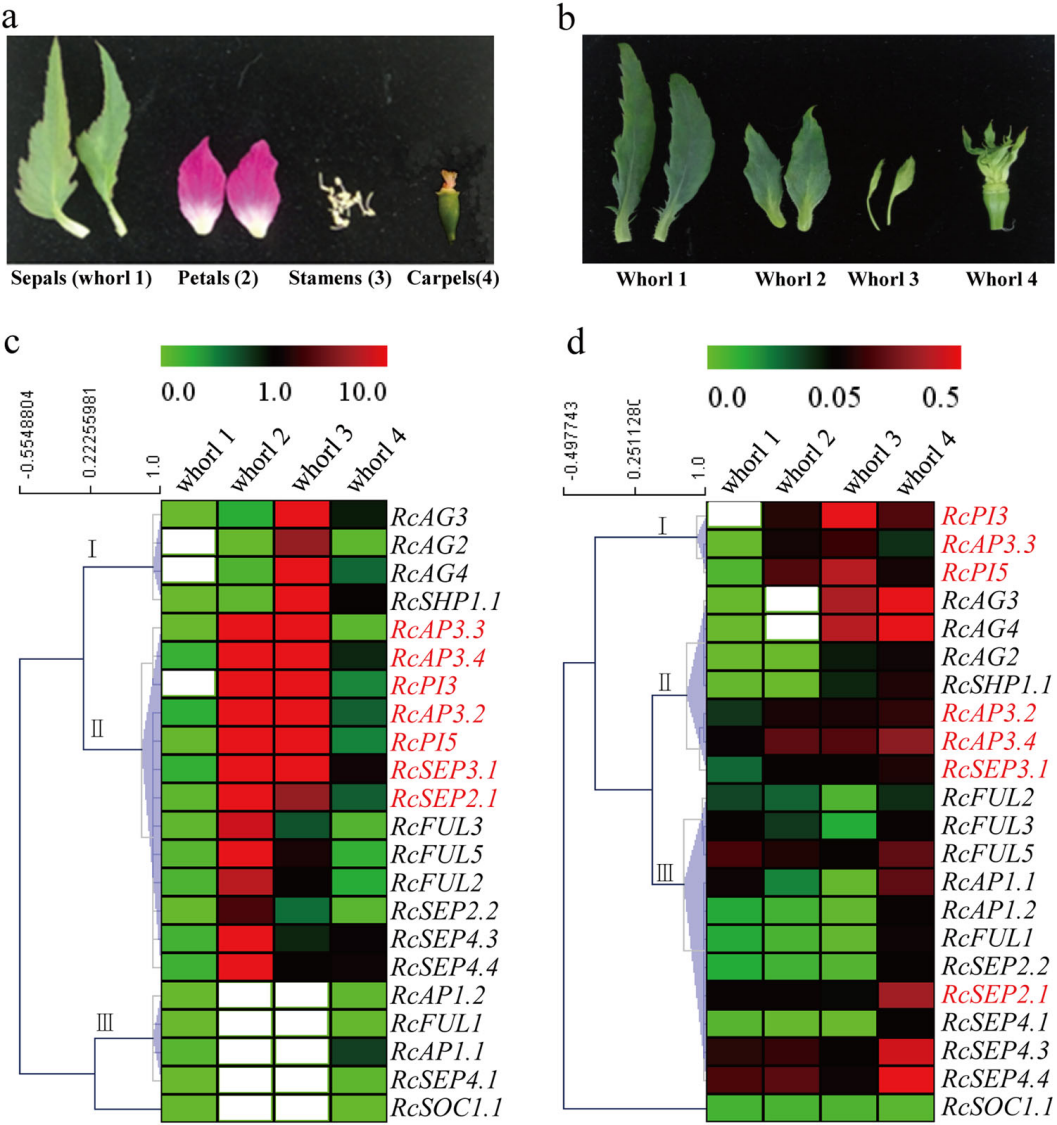
Expression patterns of MIKC^C^ genes from AP1/FUL, AP3/PI, AG, and SEP clades in different whorls of the rose flower. **a** Floral whorls of *R. chinensis* cv. Old Blush flowers, including sepals, petals, stamens and carpels. **b** Floral whorls of *R. chinensis* cv. Viridiflora. **c**,** d** Heatmaps of MIKC^C^ gene expression profiles of Old Blush and Viridiflora, respectively. The white boxes denote cases where expression was not detected

To further specify the function of the identified ABCDE model genes in floral organogenesis, the expression profiles of 21 selected rose MIKC^C^ genes (the primers of the remaining genes were not specific to our effort) from the AP1/FUL, AP3/PI, AG, and SEP clades were determined using quantitative real-time PCR (qRT-PCR) across different floral organ initiation stages of buds from Old Blush and Viridiflora, with the leaf as a control (Fig. 4c, d). The results show that almost all of the selected genes were observably up-regulated during floral organ initiation in both Old Blush and Viridiflora. However, some genes displayed different expression patterns, e.g., the expression levels of *RcFUL2*, *RcFUL3*, and *RcFUL5* were much higher in Viridiflora than in Old Blush during the floral organ initiation stages, especially the carpel initiation stage (Fig. 4c, d, stage 4). In addition, the expression of *RcAP3.3* was lost in the floral DBO and sepal initiation stages (Fig. 4d, DBO and stage 1) in Viridiflora. Lastly, *RcAP1.2*, *RcAG2* and *RcSEP4.3* were expressed only in the leaves of Viridiflora, and no expression of these genes was detected in Old Blush. The different expression profiles of these genes suggest their important roles in floral organ development in rose. Furthermore, cluster analysis of gene expression showed that both clusters II and III contained AP3/PI clade genes (Fig. 5a,b), and AG and AP1 genes were always separated into cluster II and cluster III, respectively, in both Old Blush and Viridiflora. Interestingly, four genes, i.e., *RcAP3.2*, *RcAP3.4*, *RcPI3* and *RcSEP2.1*, changed their positions from cluster III to cluster II when the floral organs mutated into leaf-like structures in Viridiflora, reflecting their specific roles in rose floral organ development.

To clarify the temporal accumulation requirement of rose AP1/FUL, AP3/PI, AG, and SEP clade genes for floral organogenesis, the fold changes of gene expression in the sepal (stage 1), petal (2), stamen (3), and carpel (4) initiation stages were calculated via comparison with the previous development stage (Fig. 4e). In Old Blush, *RcSEP4.3* (14.6-fold), *RcSEP4.4* (9.7), and *RcSHP1.1* (3.5) were strongly upregulated during sepal initiation, whereas *RcAG2* (− 5.9-fold), *RcAG3* (− 4.6), and *RcAG4* (− 7.5) were significantly downregulated. Five genes, i.e., *RcPI3* (3.8-fold), *RcAG4* (3.8), *RcSEP3.1* (3.4), *RcSEP4.4* (3.2), and *RcAP3.3* (2.9), were upregulated during petal initiation; all four genes from the AP3/PI and AG clades, and *RcSEP3.1*, *RcSEP2.1*, *RcSEP4.3* (4.5-fold), and *RcSEP4.4* (4.0) from the SEP clade strongly accumulated during stamen initiation; eight genes, i.e., *RcAP1.1* (10.2-fold), *RcAP1.2* (4.2), *RcAP3.2* (8.2), *RcAP3.4* (10.7), *RcPI3* (8.8), *RcPI5* (6.5), *RcAG3* (4.0), and *RcAG4* (3.8), were significantly increased during carpel initiation. However, when comparing the MIKC^C^ gene expression patterns of Old Blush with those of Viridiflora (Fig. 4e), we found that the accumulation states of most of the genes we surveyed in Viridiflora were dramatically changed. For example, the prominent expressions of *RcAP1.1* and *RcAP1.2* changed from the carpel-like initiation stage to the sepal- and stamen-like initiation stages, the prominent expressions of *RcAP3.2*, *RcAP3.4*, *RcPI3*, and *RcPI5* were lost during the carpel-like initiation stage and the prominent expressions of *RcSEP4.1*, *RcSEP4.3*, and *RcSEP4.4* expanded during the petal- and stamen-like initiation stages. These shifts in expression patterns not only indicate the important roles of MIKC^C^ genes in regulating the mutant floral phenotypes in Viridiflora but also highlight their potential homeotic functions in floral organogenesis and development.

### The expression profiles of AP1/FUL, AP3/PI, AG, and SEP clade genes in different whorls of rose flowers

To gain more insight into the homeotic functions of the rose homologs of A-, B-, C-, D-, and E-class genes involved in floral organogenesis, the relative expression levels of AP1/FUL, AP3/PI, AG, and SEP clade genes in different floral whorls of both Old Blush and Viridiflora were determined using qRT-PCR (Fig. 5a, b). In the normal flower genotype of Old Blush, three gene clusters (I–III) were formed based on their expression patterns (Fig. 5c). Specifically, cluster I, containing *RcAG2*, *RcAG3*, *RcAG4*, and *RcSHP1.1*, strongly accumulated in whorl 3 (stamens) and was expressed at very low or undetectable levels in the other whorls of the rose flower (the expressions of *RcAG2* and *RcAG4* were lost in whorl 1). Cluster II, which contained all of the analyzed genes from clade AP3/PI, most of the SEP clade genes (except *RcSEP4.1*), and *RcFUL2*, *RcFUL3*, and *RcFUL5* from the AP1/FUL clade, exhibited relatively higher expression levels in the second (petals) and third whorls but lower expression in the two other whorls (sepals and carpels). The expression level of B-class genes (AP3/PI clade) was remarkably higher in the second and third whorls, indicating that these rose B-class genes may be required for petal and stamen morphogenesis and development. In contrast, cluster III, which contained *RcAP1.1*, *RcAP1.2*, *RcFUL1*, and *RcAP4.1*, showed a very low expression in whorl 1 (sepals) and whorl 4 (carpels), and no expression in whorls 2 and 3, indicating the specific function of these genes in sepal and carpel morphogenesis and development.

**Fig. 6:**
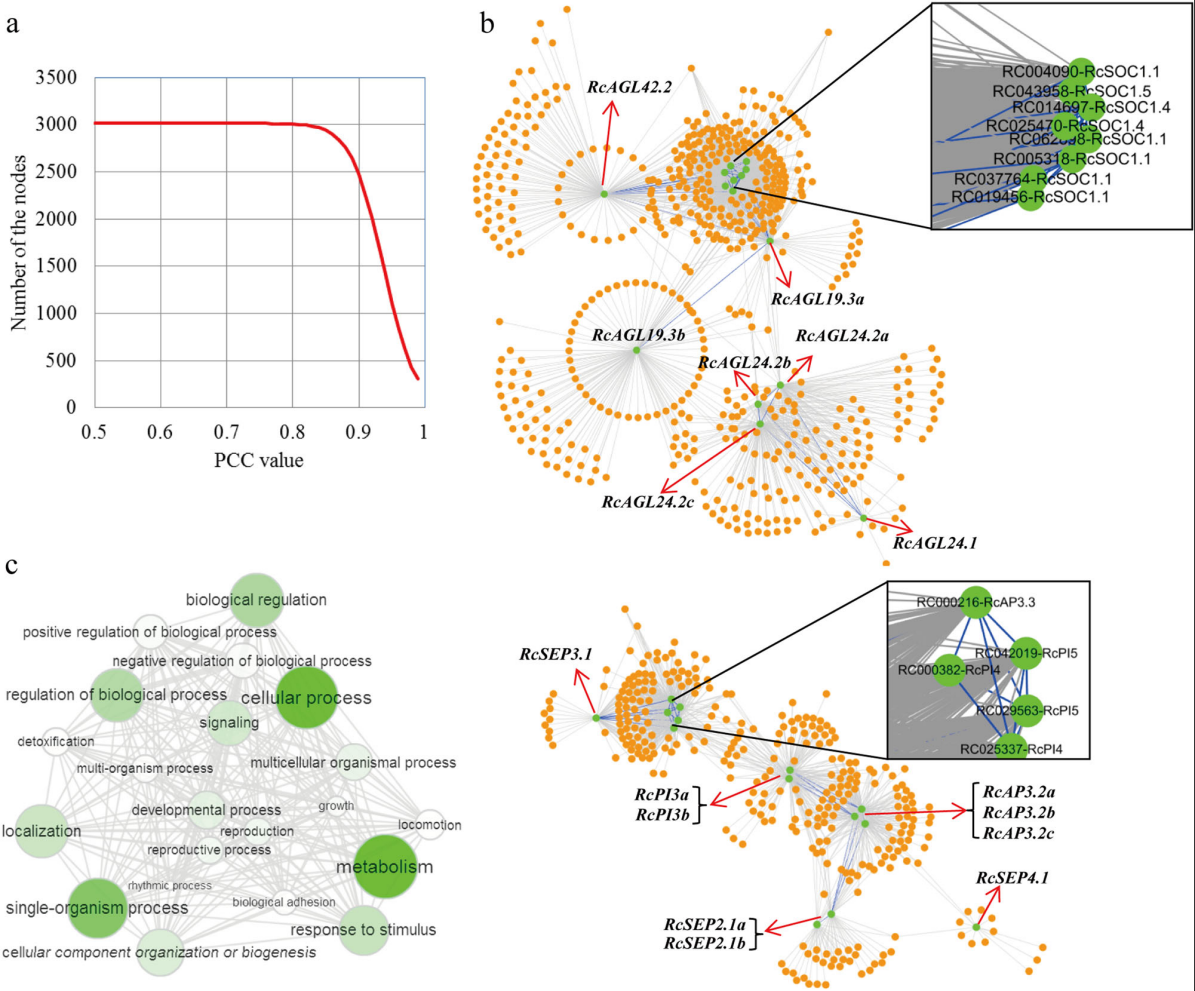
Co-expression networks of rose MIKC^C^ genes at the transcriptome level. **a** The distribution of Pearson’s correlation coefficient (PCC) values—PCC 0.77 was selected as the effective threshold in our study (see Materials and Methods section for details). **b** Co-expression networks of MIKC^C^ genes constructed at a transcriptome-wide level. MIKC^C^ genes are colored in green, and their adjacent genes are colored in orange. **c** The interactive graph of GO term enrichments of the adjacent genes from co-expression networks

Although the petals, stamens and pistils of Viridiflora have been converted into leaf-like organs, the different whorls of the floral organs can still be distinguished (Fig. 6b). A comparative analysis of gene expression in the different whorls of the floral organs of both Old Blush and Viridiflora (Fig. 6c, d) showed that the regional expression restrictions of the rose A-, B-, C-, D-, and E-class genes in the different whorls of floral organs shifted widely in Viridiflora vs. Old Blush (Fig. 5d). Specifically, the elevated expression levels of four genes, i.e., *RcAG2*, *RcAG3*, *RcAG4*, and *RcSHP1.1*, which are homologs of C/D-class genes, were extended from whorl 3 to whorl 4 in Viridiflora. Simultaneously, the expressions of *RcAG3* and *RcAG4* were lost in whorl 2 of Viridiflora, indicating their involvement in petal morphogenesis and development. Similarly, the expression superiority of *RcAP3.3*, *RcPI3*, and *RcPI5* seemed to be extended to whorl 4 from petals (whorl 2 and whorl 3). Furthermore, the expression peaks of ten genes, i.e., *RcAP3.2*, *RcAP3.4*, *RcSEP3.1*, *RcFUL2*, *RcFUL3*, *RcFUL5*, *RcSEP2.1*, *RcSEP2.2*, *RcSEP4.3*, and *RcSEP4.3*, changed dramatically from whorl 2 and whorl 3 to whorl 4 in Viridiflora compared with Old Blush, whereas *RcAP1.1*, *RcAP1.2*, *RcFUL1*, *RcSEP4.1*, and *RcSOC1.1* showed increased expression in whorl 2 and whorl 3 in Viridiflora vs. Old Blush. Interestingly, the cluster positions of seven genes (red gene names), i.e., *RcAP3.2*, *RcAP3.3*, *RcAP3.4*, *RcPI3*, *RcPI5*, *RcSEP2.1*, and *RcSEP3.1*, were changed in Viridiflora, further highlighting their role in floral organogenesis. Taken together, there data collectively demonstrate the expression patterns and important roles of A-, B-, C-, D-, and E-class genes in rose floral organogenesis.

### Co-expression networks of rose MIKC^C^ genes at the transcriptome level

To gain more insight into the regulatory relationships of rose MIKC^C^ genes, we constructed co-expression networks of all rose genes at the transcriptome level. An effective PCC threshold of 0.77 was trained to generate the lowest density networks as described in the Materials and Methods section (Fig. 6a). Finally, two independent modules were revealed for the rose MIKC^C^ genes (Fig. 6b). The first module of the network, which represented vegetative tissue development and flower transition, contained seven MIKC^C^ genes (nodes), i.e., *RcAGL19.3*, *RcAGL24.1*, *RcAGL24.2*, *RcSOC1.1*, *RcSOC1.4*, *RcSOC1.5*, and *RcAGL42.2*, and 559 other adjacent genes (nodes) and 1894 co-expression events (edges) (Fig. 6b, upper panel). All of the detected MIKC^C^ genes can be considered as hubs due to their high connectivity in the network. The frequency of co-expression events for these genes ranged from 22 to 770, and in which the edge numbers of *RcSOC1.1* (770 edges), *RcSOC1.4* (318), *RcAGL24.2* (275), and *RcAGL19.3* (261) were remarkably high, suggesting their prominent roles in vegetative tissue development and flower transition in rose. Furthermore, out of 559 other adjacent genes in the network, 234 (41.9%) had at least two connections with detected MIKC^C^ genes. Among them, 22 genes were identified as having the highest connectivity to MIKC^C^ genes with at least five edges, suggesting their important biological functions in regulating flower transition in rose.

The second module of the network, which contained eight MIKC^C^ genes (nodes), 297 other adjacent genes (nodes), and 1039 co-expression events (edges), represented reproductive tissues and flower development due to the high expression of member genes in reproductive tissues (Fig. 6b, lower panel). Interestingly, only genes from the AP3/PI and SEP clades were detected in the network, i.e., the *RcAP3.2*, *RcAP3.3*, *RcPI3*, *RcPI4*, *RcPI5*, *RcSEP2.1*, *RcSEP3.1*, and *RcSEP4.1* genes, all of which were considered to be hubs due to their high connectivity in the network. Among them, the edge number of the AP3/PI clade genes, including *RcPI3* (109 edges), *RcPI5* (99), *RcAP3.2* (94), and *RcPI4* (86), was much higher than that of the SEP clade genes, suggesting that the AP3/PI clade genes may have critical roles in regulating floral organogenesis and development in rose. Furthermore, out of 297 other adjacent genes, 158 (53.2%) had at least 2 connections with detected MIKC^C^ genes and 55 (18.5%) of them had 4 connections with detected MIKC^C^ genes, suggesting their potential roles in regulating floral development via the ABCDE model in rose.

In addition, Gene Ontology (GO) term annotations were surveyed for all the adjacent genes (859 genes) in the networks to gain more insight into their biological function bias. Among these, 260 (30.3%) genes were found to be enriched in one biological process (BP) network (Fig. 6c). The following GO categorizations were significantly enriched: biological regulation, metabolism, cellular process, and single-organism processes. More importantly, 19 genes were enriched in developmental processes and reproductive process categorizations (Fig. 6c), suggesting their potential roles in regulating floral organogenesis and development in rose. Furthermore, 118 adjacent genes were identified to be vegetative or reproductive tissue specific (Supplementary File S4).

## Discussion

The evolution of reproductive organ morphologies in seed plants, especially of floral transition and floral organogenesis in angiosperms, is considered to be a major evolutionary innovation in plant evolution and likely had a key role in Darwin’s ‘abominable mystery’^3^. MIKC^C^ genes, which were initially discovered as floral organ identity genes, originated in ancestral seed plants and have central regulatory roles in reproductive organ morphologies and flowering transition. Although MADS-box genes have been studied for approximately three decades, primarily in model plants such as *Arabidopsis*^2,3,11^, the biological functions and regulatory mechanisms of MIKC^C^ genes during flowering transition and floral organogenesis are still not fully understood, especially in Rosaceae. Comprehensive analysis of MIKC^C^ genes in rose will facilitate the understanding of plant floral organogenesis and development.

### The expansion of ABCDE model genes, and FLC and AGL17 clade genes was not detected in *R. chinensis*

In our study, 58 non-redundant MIKC^C^ unigenes were identified from rose transcriptomes based on the presence of the highly conserved MADS-box domain (Supplementary File S2). Phylogenetic analysis then divided the MIKC^C^ genes into 12 major gene clades along with their counterparts in *Arabidopsis* and strawberry^1,53^ (Fig. 1a). Interestingly, the ABCDE model and SOC1 and AGL6 clades in rose (Fig. 1b, II cluster) were found to be remarkably expanded relative to the corresponding clades in *Arabidopsis*, suggesting that enhanced and/or novel roles of the associated genes might be required for specific aspects of floral development in rose. In contrast to these expanded clades, no rose MIKC^C^ proteins are classified into the AGL17 clade observed in *Arabidopsis* and strawberry, and neither rose nor strawberry has genes in the FLC clade (Fig. 1b, I cluster), suggesting that significant gene loss might have happened in these clades after the split of *Arabidopsis* and rosaceae during their evolution. Furthermore, it is worth mentioning that the *FLOWERING LOCUS C* (*FLC*) gene and the *MADS AFFECTING FLOWERING* (*MAFs*) genes in the FLC clade act as key regulators in plant vernalization, the process that represses flowering and ensures that plants survive winter^54–57^. The non-detection of FLC clade genes in rose or strawberry could potentially indicate a re-modulation of the plant flowering mechanisms in these species^23^ and may even be associated with CF traits.

### Comparative expression analysis of AP1/FUL, AP3/PI, AG, and SEP clade genes between Old Blush and Viridiflora reveals their roles in rose floral organogenesis

Dubois et al.^43^ constructed a high-throughput gene expression atlas for rose using RNA-seq, which covered almost all the typical organs (six vegetative and seven reproductive organs) of rose plants. In total, 52 annotated unigenes were identified as MIKC^C^ genes from the unigene datasets in our study (Fig. 3a). Most of the genes from the AP1/FUL, AP3/PI, AG, and SEP clades, as well as from the AGL6 clade, were specifically expressed in the reproductive organs of rose, which is consistent with their particular roles in floral organogenesis and development^2,3,11^. Furthermore, the increase in members of the AP3/PI, AG, and SEP clades reflects the remarkable expansion of ABCDE model genes and supports their robust roles in rose floral organogenesis and development.

Furthermore, the expression profiles of genes from the AP1/FUL, AP3/PI, AG and SEP clades were extensively surveyed in multiple floral organ initiation stages in Old Blush (Fig. 4a) and the homeotic mutant Viridiflora (Fig. 4b) using qRT-PCR. The results showed that almost all of the examined genes were significantly up-regulated in response to floral organ initiation in both Old Blush and Viridiflora, suggesting roles for these genes in floral organogenesis and development in rose. The cluster analyses showed that *AG* and *AP1* genes were always separated into cluster II and cluster III, respectively (Fig. 4c,d), corresponding with the fact that A- class genes are regionally antagonistic to the expression of C-class genes in different whorls of flowers^9,58^. In addition, three genes (*RcAP3.2*, *RcAP3.4*, and *RcPI3*) from the AP3/PI clade and one gene (*RcSEP2.1*) from the SEP clade were found to have shifted cluster positions from cluster III to II, suggesting their potential roles in contributing to the floral organ mutations of Viridiflora as well as their critical roles in rose floral organogenesis and development. Subsequently, the fold changes of all of the examined genes were calculated between two adjacent floral organ differentiation stages for sepal, petal, stamen, and carpel organogenesis in rose buds (Fig. 4e). The fold changes for specific floral organ initiation of all genes from the AP3/PI clade (B- class), two genes (*RcAP1.1* and *RcAP1.1*) from the AP1/FUL clade, and three genes (*RcSEP4.1*, *RcSEP4.3*, and *RcSEP4.4*) from the SEP clade (E-class) were dramatically different between Old Blush and Viridiflora, further revealing their possible role in floral organogenesis and development.

Regional expression in different whorls of flowers is a key aspect of A-, B-, C-, D-, and E-class genes that contribute to floral organogenesis^11,12^. In our study, the regional expression restrictions of genes from the AP1/FUL, AP3/PI, AG and SEP clades were found to be dramatically changed in Viridiflora, with the expression peak of many of the genes shifted from whorl 2 and whorl 3 to whorl 4 in Viridiflora relative to Old Blush (Fig. 5). Furthermore, the expressions of *RcAP1.1*, *RcAP1.2*, *RcFUL1*, *RcSEP4.1*, and *RcSOC1.1* were extended in whorls 2 and 3 in Viridiflora, suggesting their possible important roles in rose floral organogenesis.

### Co-expression analysis further positions specific MIKC^C^ genes as hubs of the regulatory network of rose flowering development and organogenesis

Even though MIKC^C^ genes have been extensively studied in plants for almost 30 years^2,3,11^, the full nature of their position in the regulatory networks controlling flowering time and floral organogenesis remains unclear. In addition, other types of genes and factors (e.g., small RNAs, long non-coding RNA, and histone modification) have also been found to be involved in the control of floral organ structure and development^11,59–65^. In our study, co-expression networks of MIKC^C^ genes were constructed from transcriptome data using public RNA-seq data^43^. Fifteen (24.2%) out of the 58 detected MIKC^C^ genes were found to be network hubs, with 856 other adjacent genes co-expressed with them (Fig. 6b), suggesting their potential regulatory roles in controlling flowering time and floral organogenesis in rose. Interestingly, *RcAGL19*, *RcAGL24*, and *RcSOC1* were found in the vegetative module of the networks—this is surprising given work in *Arabidopsis* establishing these genes as key components of the vernalization pathway^23^, suggesting their crucial roles in regulating floral transition in rose. Interestingly, the FLC genes along with their homologs (*MAFs*) in the FLC clade that were characterized as the prolonged repressors of the vernalization pathway^23,66^ have been exhaustively lost in rose, leading us to hypothesize that the loss of genes from the FLC clade may be one of the factors contributing to CF in rose. In addition, only genes from the AP3/PI clade (*RcAP3.2*, *RcAP3.3*, *RcPI3*, *RcPI4*, and *RcPI*) and SEP clade (*RcSEP3.1* and *RcSEP4.1*) were found in the reproductive module of the networks (Fig. 6b), suggesting prominent roles for AP3/PI clade genes in regulating floral organogenesis and development in rose. This is consistent with the results from the analyses of the MADS-box domain divergence and expression patterns we described above (Fig. 2a, c, 4e and 5c, d).

In conclusion, we identified 58 non-redundant MIKC^C^ unigenes from the rose transcriptome. Subsequent analysis revealed that ABCDE model genes (corresponding to AP1/FUL, AP3/PI, AG and SEP clades), and SOC1 and AGL6 clade genes were remarkably expanded in rose. Comparative expression analysis of MIKC^C^ genes, especially the ABCDE model genes, between *R. chinensis* cv. Old Blush and the closely related homeotic mutant genotype *R. chinensis* cv. Viridiflora, confirmed their role in regulating floral organogenesis and development in rose. Furthermore, the co-expression networks provided an overview of the regulatory networks of MIKC^C^ genes and shed further light on the prominent roles of AP3/PI clade genes in floral organogenesis and the roles of *RcAGL19*, *RcAGL24*, and *RcSOC1* in regulating floral transition in rose.

## Materials and methods

### Data retrieval for the rose transcriptome

Rose transcriptome data were retrieved from public RNA-seq databases, including NCBI/SRA and GEO DataSets (https://www.ncbi.nlm.nih.gov), the DDBJ/sequence Read Archive (http://trace.ddbj.nig.ac.jp/dra/index_e.htm), GDR (https://www.rosaceae.org/), and LIPM (https://lipm-browsers.toulouse.inra.fr/plants/R.chinensis), as well as from published papers^43,48–50,67,68^. In addition, two high-quality RNA-seq datasets of *R. chinensis* cv. Old Blush and *R. chinensis* cv. Viridiflora (including four vegetative samples and nine floral organ samples) were recently generated by our own laboratory (Illumina HiSeq X-ten; Pair-end; N50 > 1463; Q30: 99.9%), and the unigene datasets were integrated into the database (unpublished). To produce a more accurate transcriptome database for rose, only three high-quality RNA-seq datasets from public databases^43,48,49^ and the two datasets produced previously by our group were selected to construct a rose transcriptome database (composed of 368,514 unigenes) for further analysis in our study (Supplementary File S5). This database included 36 samples and covered all the typical organs and their developmental stages of the rose plant.

### Transcriptomic identification of MIKC^C^ genes in rose

All sequences from our rose transcriptome database were translated in all six reading frames by a Perl script (Supplementary File S6). A hidden Markov model (HMM) search was then carried out in the six-frame protein database using two different HMM profiles. One profile was constructed by Gramzow and Theißen^32^ (Supplementary File S7). The second HMM profile used was the SRF-TF domain HMM profile (PF00319) obtained from the Pfam database (http://pfam.xfam.org/). HMM searches were executed using the HMMER3.0 software package with default thresholds (http://hmmer.org/)^69^. The protein sequences of the output unigenes were subsequently verified through public databases including SMART (http://smart.embl-heidelberg.de/) and Pfam to confirm the integrity of the MADS-box domains. Finally, the corresponding nucleotide sequences of all candidate genes were submitted to the CD-HIT Suite server, with 90% sequence identity cut-off (http://weizhong-lab.ucsd.edu/cdhit_suite/cgi-bin/index.cgi?cmd=Server%20home) to remove redundant sequences along with manual checking (Supplementary File S8). Finally, MIKC^C^ genes were separated from all identified MADS-box genes by further phylogenetic analysis. The same protocols were performed for the identification of MADS-box genes in the strawberry (*Fragaria vesca*) genome (Supplementary File S9).

### Sequence alignment and phylogenetic analysis

Multiple sequence alignment was performed via the alignment software MAFFT v7.037b^70^ using the accurate E-INS-I alignment strategy, which is stated to be suitable for sequences containing large unalignable regions. For the phylogenetic analysis, accurate maximum-likelihood trees were constructed using FastTree software with default parameters (http://www.microbesonline.org/fasttree/)^71^. The phylogenetic tree was visualized using MEGA7 (http://www.megasoftware.net/home)^72^. The tertiary structures and homo-/heterodimeric states of MADS-domain proteins were predicted using the SWISS-MODEL online program (https://swissmodel.expasy.org/interactive)^73^.

### Plant materials and sample collection

Two-year-old cutting seedlings of *R. chinensis* cultivars, Viridiflora, and Old Blush (both are Chinese old roses), were grown in a greenhouse. Flower buds were collected with stems carrying two leaves and were dipped directly into RNase-free water. The flower buds were then vertically dissected into two equal parts under a stereo microscope (Fluorescent Stereo Microscope Leica M165 FC, Wetzlar, Germany) to confirm accurate determination of the stages of floral organ differentiation. The validated samples were immediately placed into liquid nitrogen and stored at − 80 ℃ until RNA extractions. This procedure was completed for each sample in < 2 min.

### RNA isolation and qRT-PCR determination

Total RNAs of samples were extracted using the BioTeke Quick RNA Isolation Kit (Catalog number: RP3301, BioTeke Corporation, Beijing, China) according to the manufacturer’s instructions. High-quality total RNA (1 μg) was reverse transcribed using the PrimeScript^TM^ RT Reagent Kit with gDNA Eraser (Perfect Real Time) (catalog number: RR047A, TaKaRa, Dalian, China) according to the manufacturer’s instructions. Gene-specific primers for MIKC^C^ genes were designed using GenScript (https://www.genscript.com/) and the IDT (http://sg.idtdna.com/scitools/Applications/RealTimePCR/) online server. qRT-PCR assays were carried out by the QuantStudio 6 Real-Time PCR System (Thermo Fisher Scientific, California, USA) using SYBR^®^ Premix Ex Taq^TM^ (Tli RNaseH Plus) (catalog number: RR420A, TaKaRa) according to the manufacturer’s instructions. Expression levels were normalized with the *RcTUBULIN*, *RcGAPDH*, and *RcTCTP* reference genes, and the 2^–^^ΔCT^ method was used for calculating the relative expression of rose MIKC^C^ genes. Three biological replicates with three qPCR technical replicates were performed for each experiment. Heatmaps were generated using Mev v4.9.0 software (http://www.tm4.org/), and the Pearson’s correlation distance was used for cluster analysis. The sequences of primers are available in Supplementary File S10 and their melting curve analyses are presented in Supplementary File S11.

### Co-expression networks

Co-expression networks were generated by using the RNA-seq datasets of rose downloaded from LIPM (https://lipm-browsers.toulouse.inra.fr/plants/R.chinensis)^43^, which covered 13 tissues (6 vegetative and 7 reproductive samples) of rose plants. First, *t*-tests were performed to calculate the significance level of all unigenes between vegetative and reproductive samples based on gene expression (FPKM values). A total of 3014 genes differed significantly between vegetative and reproductive samples, with a *p*-value of < 0.05 (Supplementary File S12). Then, the R package was employed to determine the Pearson’s correlation coefficient (PCC) values between all selected unigenes. An effective threshold (0.77) of the PCC value was trained to generate the lowest density networks using R script (Supplementary File S13). Finally, the MIKC^C^ genes (nodes) with their adjacent connections (edges) were extracted for our analysis (Supplementary File S14). The constructed co-expression networks were visualized using the Cytoscape v. 3.5.1 program (http://www.cytoscape.org/)^74^. GO term enrichment was performed using Blast2GO software (https://www.blast2go.com/) and was visualized using REVIGO (http://revigo.irb.hr/)^75^.

## Electronic supplementary material

Supplementary File S1 The flower phenotypes of Rosa chinensis cv. Old Blush (a, c) and Viridiflora (b, d). (e and f) present the almost identical leaves of Old Blush (e) and Viridiflora (f), respectivel(TIF 1075 kb)

Supplementary File S2. MIKC type MADS-box genes identified from Rosa chinensis transcriptome(XLSX 19 kb)

Supplementary File S3. The homo-/heterodimeric state of the MADS-box domain proteins form Arabidopsis, strawberry and rose(XLSX 13 kb)

Supplementary file S4. The vegetative or reproductive tissue-specific genes in the co-expression networks(XLSX 18 kb)

Supplementary File S5. The integrated transcriptome database of Rosa chinensis(TXT 277657 kb)

Supplementary File S6. Perl script for translating the nucleotide sequences of rose RNA-seq unigenes into six reading frames proteins(TXT 3 kb)

Supplementary File S7. The hidden Markov model (HMM) of MADS-box domain(TXT 22 kb)

Supplementary File S8 The redundant genes removed by CD-HIT Suite server(TXT 8 kb)

Supplementary File S9. The protein sequences of MADS-box genes identified from strawberry (Fragaria vesca) genome(TXT 69 kb)

Supplementary File S10. The primers of MIKCC genes used for qRT-PCR assay(XLSX 17 kb)

Supplementary File S11. The melt curve plots of primers used for qRT-PCR assay(PDF 1206 kb)

Supplementary File S12. Total 3014 genes that varied significant between vegetative and reproductive tissues in rose transcriptome(XLSX 317 kb)

Supplementary File S13. The R script of training the appropriate PCC threshold for the co-expression networks(TXT 1 kb)

Supplementary File S14. The co-expression networks of MIKCC genes for Cytoscape visualization(ZIP 119 kb)
