# Supplementary figures and images for: MIKCC-type MADS-box genes in Rosa chinensis: the remarkable expansion of ABCDE model genes and their roles in floral organogenesis
Source: Hortic Res. 2018 May 1;5:25. doi: 10.1038/s41438-018-0031-4 (PMC5928068; doi:10.1038/s41438-018-0031-4)

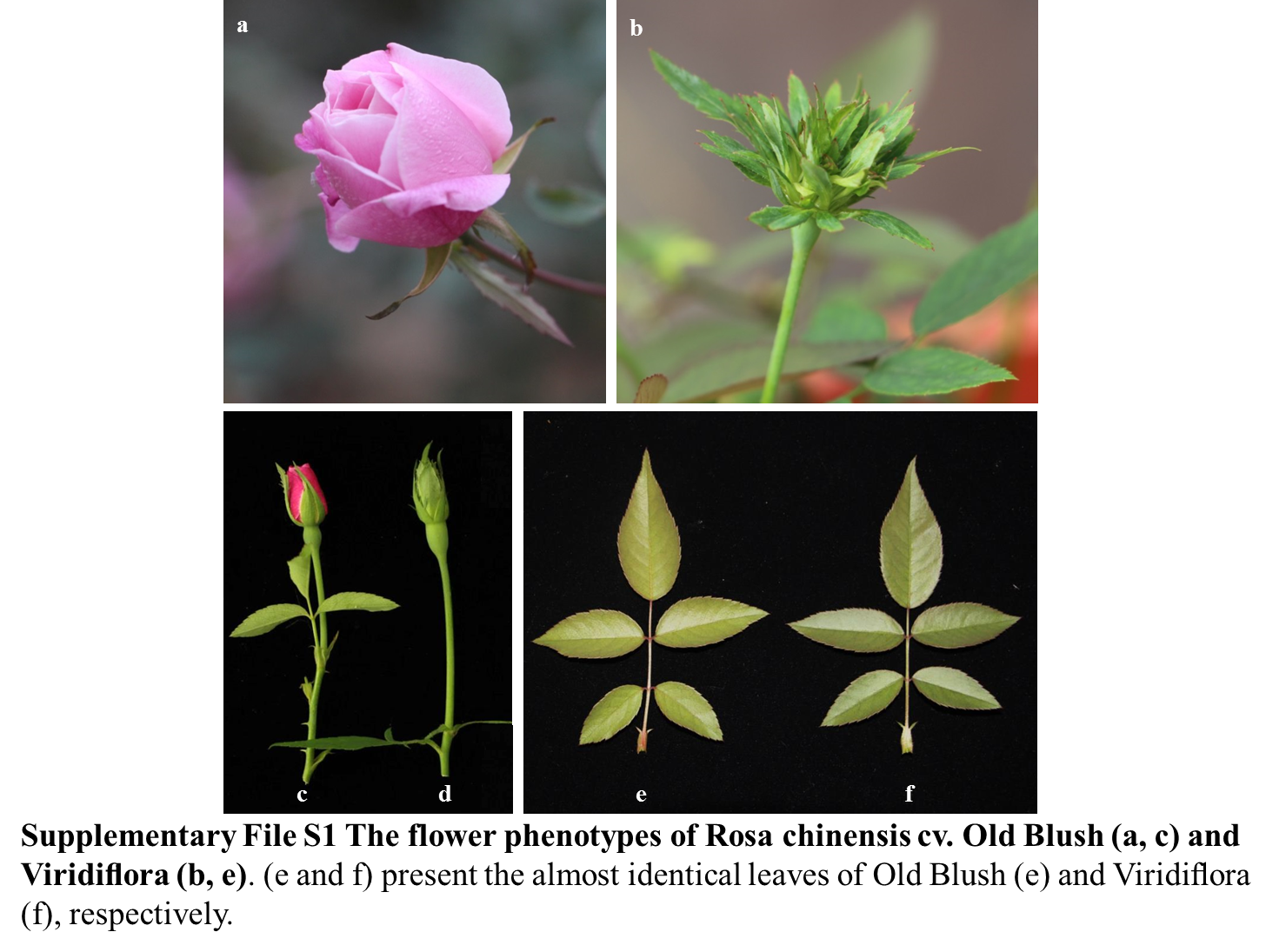

Supplement: Supplementary file 1 — Supplementary File S1 The flower phenotypes of Rosa chinensis cv. Old Blush (a, c) and Viridiflora (b, d). (e and f) present the almost identical leaves of Old Blush (e) and Viridiflora (f), respectivel(TIF 1075 kb) [file 41438_2018_31_MOESM1_ESM.tif]
