# Supplementary material for: MIKCC-type MADS-box genes in Rosa chinensis: the remarkable expansion of ABCDE model genes and their roles in floral organogenesis
Source: Hortic Res. 2018 May 1;5:25. doi: 10.1038/s41438-018-0031-4 (PMC5928068; doi:10.1038/s41438-018-0031-4)
Supplement: Supplementary file 11 — Supplementary File S11. The melt curve plots of primers used for qRT-PCR assay(PDF 1206 kb) [file 41438_2018_31_MOESM11_ESM.pdf]

Supplementary file S11  The melt curve plots of primers used for qRT-PCR assay

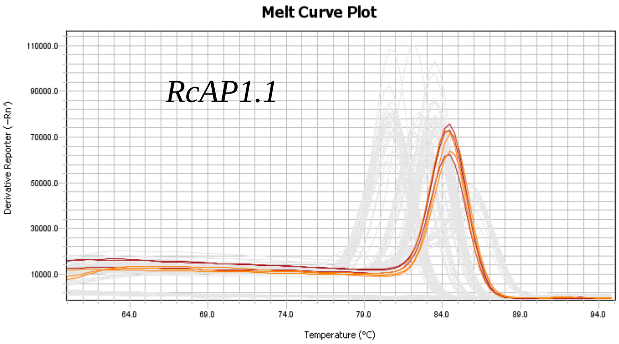


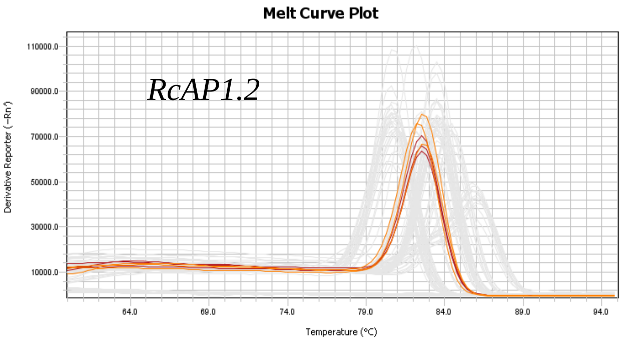


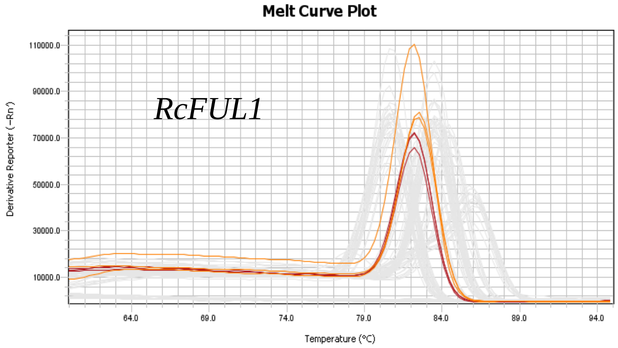


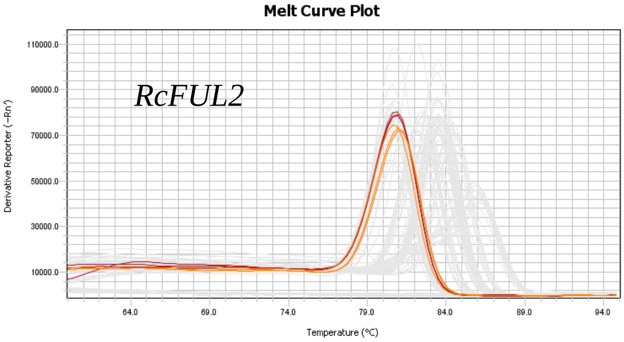


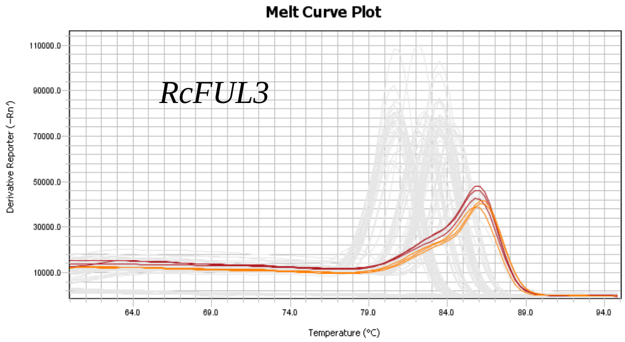


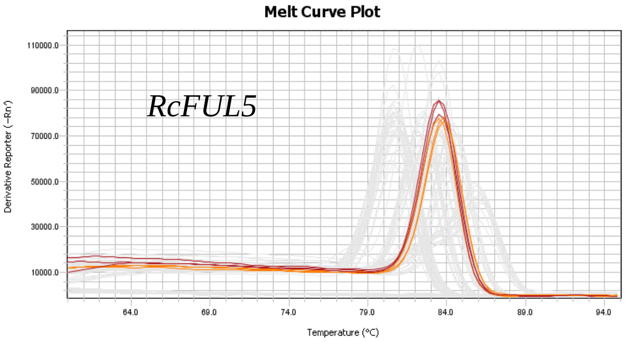


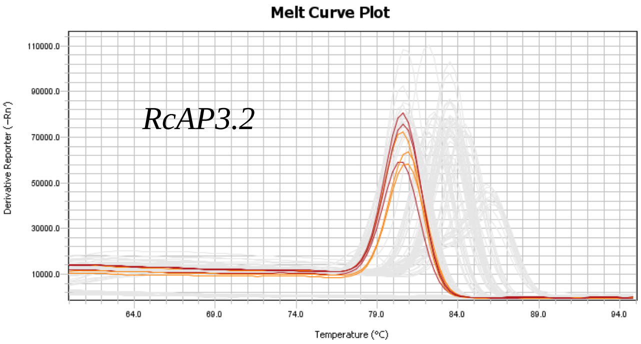


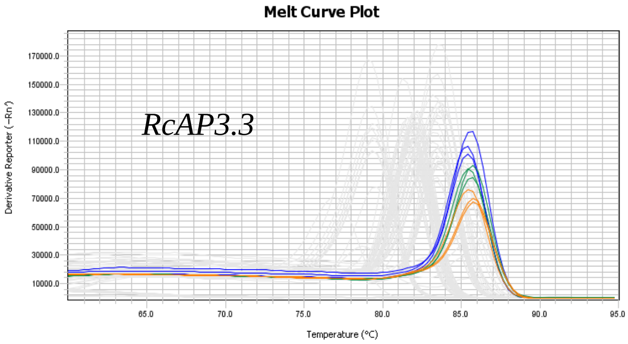


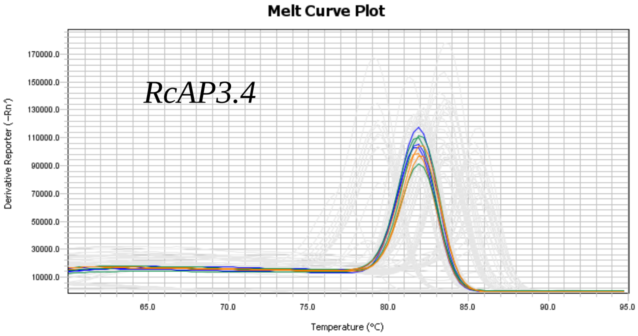


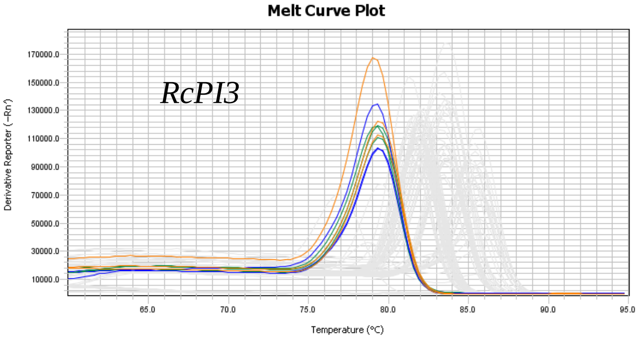

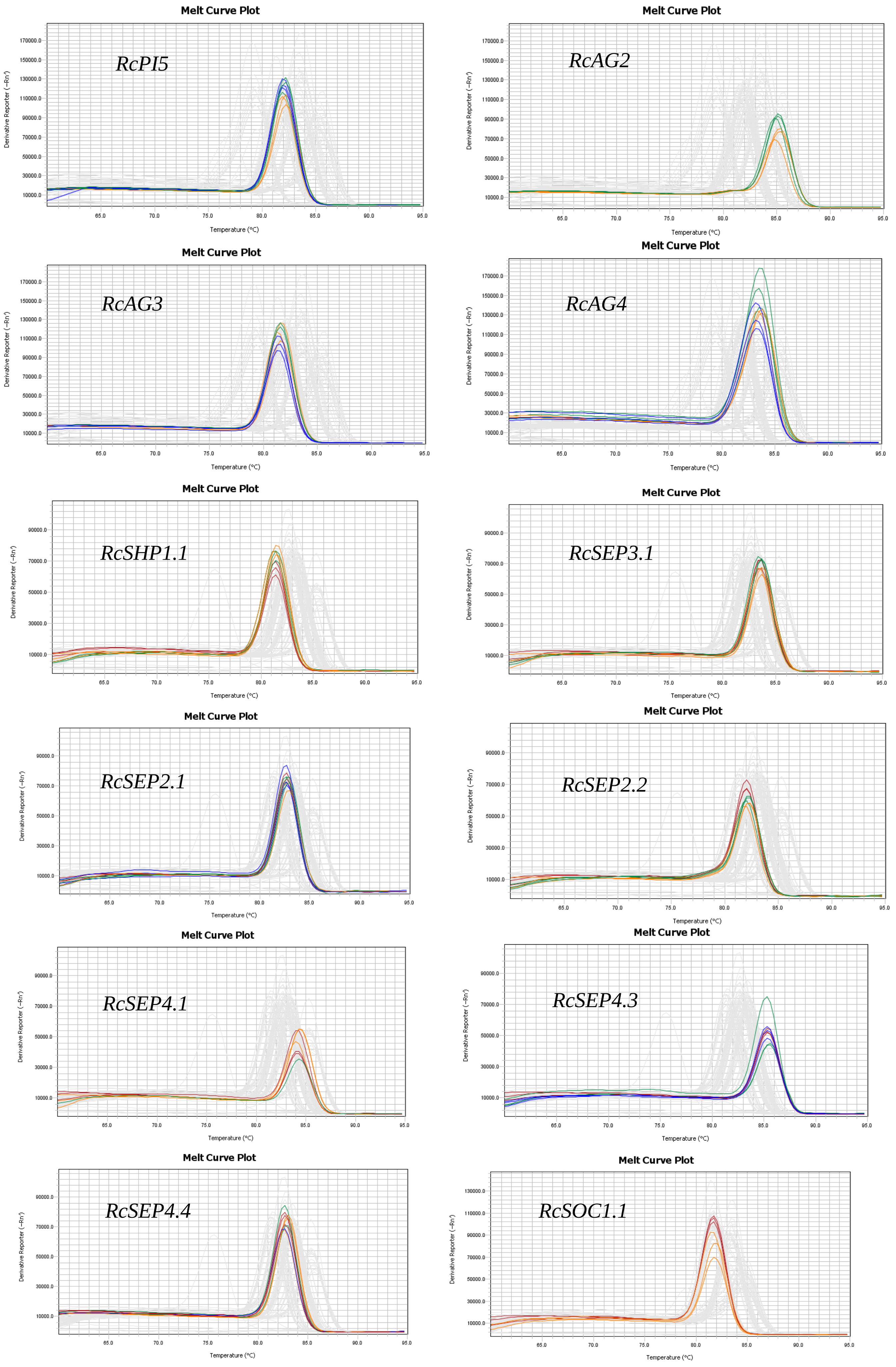

Melt Curve Plot
RcPI5
Melt Curve Plot
RcAG2
Melt Curve Plot
RcAG3
Melt Curve Plot
RcAG4
Melt Curve Plot
RcSHP1.1
Melt Curve Plot
RcSEP3.1
Melt Curve Plot
RcSEP2.1
Melt Curve Plot
RcSEP2.2
Melt Curve Plot
RcSEP4.1
Melt Curve Plot
RcSEP4.3
Melt Curve Plot
RcSEP4.4
Melt Curve Plot
RcSOC1.1
Derivative Reporter (−Rn')
Temperature (°C)
